# Oxygen Uptake Kinetics and Time Limit at Maximal Aerobic Workload in Tethered Swimming

**DOI:** 10.3390/metabo13070773

**Published:** 2023-06-21

**Authors:** Danilo A. Massini, Mário C. Espada, Anderson G. Macedo, Fernando J. Santos, Eliane A. Castro, Cátia C. Ferreira, Ricardo A. M. Robalo, Amândio A. P. Dias, Tiago A. F. Almeida, Dalton M. Pessôa Filho

**Affiliations:** 1 Graduate Programme in Human Development and Technology, Institute of Biology, São Paulo State University (UNESP), Campus at Rio Claro, São Paulo 13506-900, Brazil; dmassini@hotmail.com (D.A.M.); andersongmacedo@yahoo.com.br (A.G.M.); eliane.castro@unesp.br (E.A.C.); tiagofalmeida.w@gmail.com (T.A.F.A.); dalton.pessoa-filho@unesp.br (D.M.P.F.); 2 Physical Education Department, School of Sciences (FC), Universidade Estadual Paulista (UNESP), Campus at Bauru, São Paulo 17033-360, Brazil; 3 Instituto Politécnico de Setúbal, Escola Superior de Educação, 2914-504 Setúbal, Portugal; fernando.santos@ese.ips.pt (F.J.S.); catia.ferreira@ese.ips.pt (C.C.F.); ricardo.robalo@ese.ips.pt (R.A.M.R.); 4 Life Quality Research Centre (LQRC—CIEQV, Leiria), Complexo Andaluz, Apartado, 2040-413 Rio Maior, Portugal; 5 CIPER, Faculdade de Motricidade Humana, Universidade de Lisboa, 1499-002 Cruz Quebrada, Portugal; 6 Faculdade de Motricidade Humana, Universidade de Lisboa, 1499-002 Cruz Quebrada, Portugal; 7 LFE Research Group, Universidad Politécnica de Madrid (UPM), 28040 Madrid, Spain; 8 Research Group in Optimization of Training and Sport Performance (GOERD), Faculty of Sports Sciences, University of Extremadura, 10003 Caceres, Spain; 9 Egas Moniz Center for Interdisciplinary Research (CiiEM), Egas Moniz School of Health & Science, 2829-511 Caparica, Portugal; adias@egasmoniz.edu.pt

**Keywords:** conditioning assessment, exercise domain, oxygen uptake kinetics, tethered swimming

## Abstract

This study aimed to apply an incremental tethered swimming test (ITT) with workloads (WL) based on individual rates of front crawl mean tethered force (Fmean) for the identification of the upper boundary of heavy exercise (by means of respiratory compensation point, RCP), and therefore to describe oxygen uptake kinetics (VO_2_k) and time limit (t_Lim_) responses to WL corresponding to peak oxygen uptake (WLVO_2peak_). Sixteen swimmers of both sexes (17.6 ± 3.8 years old, 175.8 ± 9.2 cm, and 68.5 ± 10.6 kg) performed the ITT until exhaustion, attached to a weight-bearing pulley–rope system for the measurements of gas exchange threshold (GET), RCP, and VO_2peak_. The WL was increased by 5% from 30 to 70% of Fmean at every minute, with Fmean being measured by a load cell attached to the swimmers during an all-out 30 s front crawl bout. The pulmonary gas exchange was sampled breath by breath, and the mathematical description of VO_2_k used a first-order exponential with time delay (TD) on the average of two rest-to-work transitions at WLVO_2peak_. The mean VO_2peak_ approached 50.2 ± 6.2 mL·kg^−1^·min^−1^ and GET and RCP attained (respectively) 67.4 ± 7.3% and 87.4 ± 3.4% VO_2peak_. The average t_Lim_ was 329.5 ± 63.6 s for both sexes, and all swimmers attained VO_2peak_ (100.4 ± 3.8%) when considering the primary response of VO_2_ (A_1′_ = 91.8 ± 6.7%VO_2peak_) associated with the VO_2_ slow component (SC) of 10.7 ± 6.7% of end-exercise VO_2_, with time constants of 24.4 ± 9.8 s for A_1′_ and 149.3 ± 29.1 s for SC. Negative correlations were observed for t_Lim_ to VO_2peak_, WLVO_2peak_, GET, RCP, and EEVO_2_ (r = −0.55, −0.59, −0.58, −0.53, and −0.50). Thus, the VO_2_k during tethered swimming at WLVO_2peak_ reproduced the physiological responses corresponding to a severe domain. The findings also demonstrated that t_Lim_ was inversely related to aerobic conditioning indexes and to the ability to adjust oxidative metabolism to match target VO_2_ demand during exercise.

## 1. Introduction

Swimming tethered by an inelastic wire attached to a resistance, which prevents swimmer displacement in water, has offered a realistic condition to simulate unimpeded swimming (i.e., free condition) [1], therefore enabling the measurements of force during stroke (arms) and kicking (legs) movements [2,3,4,5] as well as the assessment of the physiological responses while simulating incremental or constant exercise modes [6,7,8,9]. From the physiological assessments, the results have demonstrated similarities between tethered and unimpeded swimming conditions with regard to the responses of cardio-circulatory [10] and respiratory [6,11,12] systems, blood lactate concentration [7], and energetics contribution [8].

In spite of these physiological findings, tethered swimming would still need to demonstrate reliability in contextualizing the physiological information during different levels of loads applied to graded swimming intensity, thus ensuring it is validated as an ergometer. In the context of incremental exercise, tethered swimming has been considered a reliable ergometer to ensure the temporal resolution between breath-by-breath pulmonary gas exchange response and loading management during a ramp test, which was designed to define the exercise domains from the assessment of gas exchange threshold (GET), respiratory compensation point (RCP), and peak oxygen uptake (VO_2peak_) [9,13]. In addition, these studies also demonstrated the representativeness of the VO_2_ response to different load stimuli based on individual references of maximal tethered force.

In contrast, in the context of constant exercise, there is a lack of information to support the physiological description of rest-to-work transition during tethered swimming, which might be useful to provide the necessary metabolic adjustment to reach muscular energy requirements, as has been observed by means of VO_2_ kinetics (VO_2_k) in unimpeded swimming for the characterization of exercise domains [14,15], performance in distance races [16,17,18], exercise tolerance (i.e., time limit) in continuous [19,20,21,22,23] and intermittent trials [24,25], and comparisons to other exercises modes [26]. In fact, there are findings comparing constant exercise performance and blood-lactate response during tethered to unimpeded swimming conditions [7], but the VO_2_k was not analyzed and therefore not compared. Hence, unsupported by VO_2_k analysis, the inferences on the respiratory (i.e., gas diffusion), circulatory (i.e., blood perfusion), and metabolic (i.e., aerobic and anaerobic energy sources) responses during tethered swimming are insufficient to recognize whether the underlying physiological process determining muscle tolerance, or its limitations in relation to metabolic acidosis, is not different to the well-described mechanisms for unimpeded swimming.

Therefore, the current study aimed to contribute to the validity of the physiological responses during tethered swimming conditions by defining the severe domain from measurements of pulmonary gas exchange during an incremental ramp test. An additional purpose was to confirm the isocapnic zone boundaries during an incremental ramp test, and hence distinguish the sustainable exercise zone from that associated with fatigue events of metabolic acidosis. The hypothesis was that the profile of VO_2_ kinetics supports the speculation that time limit and metabolic responses while swimming in tethered conditions assure correspondence with the established physiological responses underlying muscle fatigue in the unimpeded severe domain of swimming. Furthermore, once this speculation is confirmed, it shall be possible to emphasize the specificity of tethered swimming for characterizing the physiological responses determining exercise tolerance in the severe domain.

## 2. Materials and Methods

### 2.1. Participants

The eleven male (18.0 ± 4.0 years old, 180.2 ± 6.8 cm height, and 71.8 ± 9.5 kg body weight) and five female (16.8 ± 3.6 years old, 166.2 ± 5.5 cm height, and 61.1 ± 9.8 kg body weight) were all swimmers with at least three years of training. The training plan just before the period of assessment was 31.8 ± 10.9 km per week, which was scheduled with aerobic (64 ± 12%), anaerobic (11.5 ± 4.7%), and other (24.4 ± 8.2%) units throughout the baseline period (14 weeks). Their best unimpeded front crawl performances at 200 m (i.e., a typical middle-distance race) represented 576 ± 136 vs. 504 ± 107 FINA points for male and female swimmers (respectively).

All subjects (and their parents/guardians when <18 years old) received information on the procedures and signed an informed consent form to participate in the study. All research procedures were conducted in accordance with the Declaration of Helsinki, and previously approved by the local University Ethics Committee (CAEE: 02402512.7.0000.5398).

### 2.2. Experimental Design

To reduce drafting and pacing effects, all swimming tests were performed with no other swimmer(s) in the same or nearby lanes. Swimmers visited the swimming pool to test the maximum force in tethered swimming conditions. After 48 h, the swimmers performed the incremental tethered test (ITT), and thereafter two other tests at constant load, corresponding to the workload (WL) at VO_2peak_ (i.e., WLVO_2peak_), were performed 48 h after the initial ITT and between each other.

All tests were performed at the same period of the day to avoid circadian interference, in a 25 m swimming pool with controlled water temperature at 28 °C. All procedures were performed in the preparatory phase of the competitive season, and each swimmer concluded the entire protocol in two weeks. A familiarization period with tethered swimming and snorkel apparatus was accomplished before the tests, following previous recommendations [16,24]. The swimmers were instructed to avoid high-intensity training sessions at least 24 h before the testing, to retain their regular nutritional habits, and to avoid alcohol and/or stimulant beverages. The dietary routine was recommended to be unchanged during the experimental analysis.

### 2.3. Maximal Force Testing in Tethered Swimming

The force produced during tethered swimming was measured with a 500 kgf load cell attached to the swimmers by an inelastic rope. The load cell was previously calibrated for 100 Hz signal acquisition, with smoothing performed by the manufacturer’s software package (N2000PRO, Cefise^®^, São Paulo, Brazil). Swimmers performed the full front crawl style, trying to displace the body forward as strongly as possible (unsuccessfully) for 30 s (e.g., an all-out bout) for the analysis of force (e.g., mean peaks of force in the 30 s, Fmean), following previous recommendations [9,13]. In summary, these authors [9,13] suggested to consider a baseline (e.g., the force required to align the swimmer horizontally in water and extend the rope system with minimal strain, which should be measured just before the onset of the all-out bout) for the measurement of Fmean. The fractions of Fmean were the WL applied to grade the swimming intensity during each stage of the ITT.

### 2.4. Incremental Tethered Test (ITT)

Swimmers performed the ITT until voluntary exhaustion attached to a weight-bearing pulley rope system. As previously recommended [9,13], the swimmers were instructed to administer the front crawl with a propelling force to avoid being pulled back or forward as the WL was applied from 30% of Fmean (i.e., =(100%Fmean − baseline load) × 0.3), with increments of 5% per minute. Pulmonary gas exchange was analyzed breath by breath by a portable and automatized metabolic unit (CPET K4b2, Cosmed, Rome, Italy) coupled to a specific snorkel designed and validated for swimming (Cosmed new-AquaTrainer^®^, Rome, Italy) [27]. Prior to each test, the metabolic unit was calibrated following manufacturer recommendations, and swimmers rested for 10 min by sitting on the edge of the pool for VO_2_ baseline assessment with the snorkel system.

The breath-by-breath data were smoothed and exported in consecutive 9 s binary averages, and VO_2peak_ was achieved by a well-motivated swimmer by assessing the highest three point rolling average VO_2_ achieved in spite of the increase in WL [9,13]. The exhaustion during ITT, and consequently the end of the test, was considered the moment during which the propelling force was no longer enough to avoiding swimmers being pulled back, or keep (at least) the head inside the recommended area, despite verbal encouragement. A blood sample (25 µL) was collected in the first minute after the end of the ITT for the analysis of blood lactate concentration ([La^−^]) just after the exercise (YSL, 2300 STAT, Yellow Springs, OH, USA).

Two researchers assessed the GET and RCP by analyzing the 9 s binary averages for the responses of V_E_/VCO_2_, V_E_/VO_2_, PetCO_2_, and PetO_2_ during ITT. The criteria for GET determination were (1) increase in VE/VO_2_ and PETO_2_ and (2) no concomitant changes in V_E_/VCO_2_ and PetCO_2_ responses, under moderate misalignment between VCO_2_ and VE (hyperventilation) with WL increasing [28,29]. Therefore, GET should demarcate the point during ITT at which VE changes and the VCO_2_ increases (due to the consequent buffering of metabolic acidosis), which can be observed by an increase in the ratio of both VCO_2_ and VE to VO_2_ that causes end-tidal O_2_ to increase [9]. In turn, the RCP criteria were (1) sustained increase in V_E_/VO_2_ and V_E_/VCO_2_, (2) decreased PetCO_2_, and (3) marked hyperventilation process [29]. The WLs corresponding to GET and RCP were defined as the WL just before the step where these thresholds were observed, and the WL corresponding to VO_2peak_ was the lowest step eliciting no further increases in the VO_2_ response (see text above about VO_2peak_ assessment), which were described as WLGET, WLRCP, and WLVO_2peak_, respectively. The stroke rate (SR) was calculated using the equation (SR = 60/stroke duration) and expressed in cycles per minute (cycles.min^−1^).

Heart rate (HR) was recorded with a Polar^®^ sensor (Kempele, Finland) designed for the new-AquaTrainer^®^ system and sampled in synchronization with breath-by-breath measurements.

### 2.5. Analysis of VO_2_ Kinetics during Exercise

Two rest exercise transitions at iVO_2max_ were performed until voluntary exhaustion, following the same criteria described above for the characterization of exhaustion in the ITT. The VO_2_ samples from both transitions were time-aligned, the noise was excluded from each data set, and the transitions for each subject were interpolated second by second to obtain an average, as suggested by Özyener et al. [30]. Since time to exhaustion was not the same when comparing both transitions, the sets of transition values were equalized by the lower time performance at WLVO_2peak_, which was considered for the analysis of VO_2_k. The highest tolerance (time) obtained was considered as the time limit (t_Lim_). Blood was sampled in the first minute after the end of each transition for [La^−^] analysis (following the procedures described above for the ITT).

The mathematical description of VO_2_k was performed using the residual model from the mono-exponential adjustment with no time delay (TD) response, as previously suggested [31]. Residual analysis was applied to the delimitation of the primary component of VO_2_, limiting it to the occurrence of the slow component (SC) if it was discernible (i.e., the time period during which there was a difference between the observed and predicted VO_2_ values, after a period in which they have not successively differed) (Equation (1)) [30]. Subsequently, another mono-exponential with TD (TD_1_ in Equation (2)) was applied to describe the primary component and to obtain the time constant (τ_1_) and the amplitude of VO_2_ (A_1_). In Equation (2), the cardio-dynamic component was not considered by eliminating the initial 20 s of the VO_2_ response to exercise.

(1) $$VO_{2}\left(t\right)=VO_{2b}+A_{1}\left[1-l^{-t/\tau }\right]$$

(2) $$VO_{2}\left(t\right)=VO_{2b}+A_{1}\left[1-l^{-\left(t-TD_{1}/\tau_{1}\right)}\right]$$

where VO_2b_ is the baseline of VO_2_ (i.e., the 10 min averaged value in resting condition before each transition). The physiologically relevant increase in VO_2_ is the amplitude of the primary component (A_1′_), which should strictly reflect the kinetics of O_2_ extraction by skeletal muscle (i.e., A_1_–VO_2b_). The SC amplitude was defined as the algebraic difference between VO_2_ at the time delay of SC occurrence (TD_2_) and the value at the end of exercise (EEVO_2_, last 15 s averaged VO_2_), as measured by Equation (3).

(3) $$VO_{2}\left(t\right)=VO_{2b}+A_{1}\left[1-l^{-\left(TD_{2}-TD_{1}/\tau_{1}\right)}\right]$$

The oxygen deficit (O_2_df) during primary amplitude response was calculated according to Whipp et al. [32] as O_2_df = A_1·_MRT (with MRT—mean response time—calculated from TD_1_ and τ_1_ obtained in Equation (1)).

### 2.6. Statistical Analysis

The values were represented as mean and standard deviation, and were checked for normality by the Shapiro–Wilk test. The adjustments of VO_2_ and SR with the WL were performed based on the least-squares method, as well as the mono-exponential functions, with and without TD for the analysis of VO_2_k. The coefficient of variance (R^2^) was applied to analyze the level of association between the responses of VO_2_ and SR with the WL during the ITT. The independent *t*-test verified whether ITT and the constant load test were different with regard to the physiological response by comparing VO_2peak_ vs. EEVO_2_, as well as [La^−^] responses. Pearson’s coefficient (r) correlated t_Lim_ with the aerobic conditioning variables (VO_2peak_, WLVO_2peak_, GET, WLGET, RCP, and WLRCP), as well as with the parameters of VO_2_k (τ_1_, A_1′_, SC and O_2_df) and [La^−^] for the analysis of how aerobic conditioning indexes and metabolism responses are related to tolerance. Statistical and mathematical analyses were performed using SPSS 26.0^®^ (SPSS. Inc., Chicago, IL, USA) and OriginPro 8^®^ (Northampton, MA, USA), and the significance level was set at *p* ≤ 0.05. The sample power was determined with G*Power 3 from data including the Pearson coefficient for the observed correlation to t_Lim_, actual Newton (N) sample, and specifying α = 0.05 [33,34].

## 3. Results

**Figure 1:**
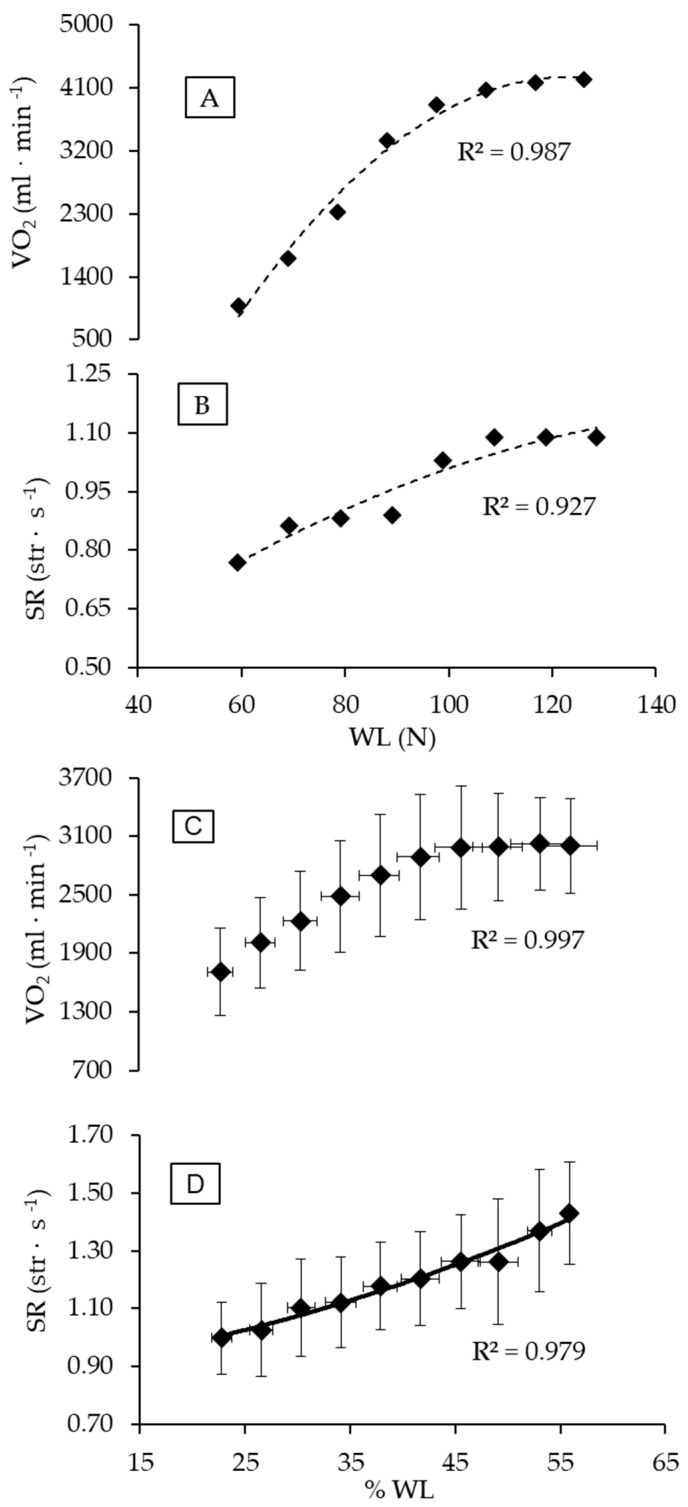
Individual (**A**,**B**) and group (**C**,**D**) profiles of VO_2_ and SR with WL increasing during ITT. The best adjustments were (**A**) VO_2_ = −0.7857x^2^ + 196.89x − 8076.9; (**B**) SR = −0.00003x^2^ + 0.0113x + 0.2147; (**C**) VO_2_ = −1.5017x^2^ + 158.18x − 1135.5; and (**D**) SR = 0.0001x^2^ + 0.004x + 0.8607. Abbreviations: SR, stroke rate; WL, workload; and ITT, incremental tether test.

**Figure 2:**
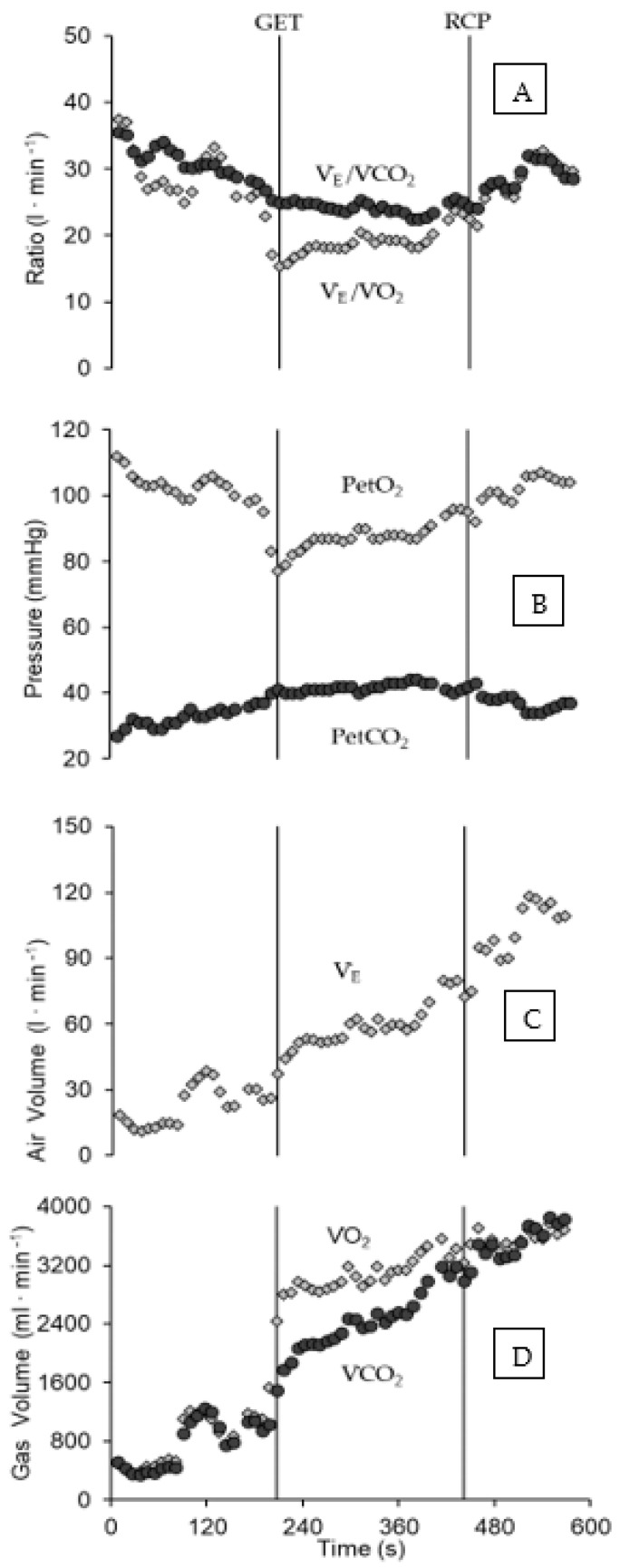
Gas exchange response during the ITT, demarcating GET and RCP (vertical lines) occurrence for a male swimmer, in accordance with the criteria for the assessment of each threshold. The Panels are depicting the profiles for V_E_/VCO_2_ and V_E_/VO_2_ vs. time (**A**), PetO_2_ and PetCO_2_ vs. time (**B**), V_E_ vs. time (**C**), and VO_2_ and VCO_2_ vs. time (**D**), Abbreviations: GET (gas exchange threshold), RCP (respiratory compensation point), PetCO_2_ (end-tidal pressure CO_2_), PetO_2_ (end-tidal pressure O_2_), VO_2_ (O_2_ uptake), VCO_2_ (CO_2_ output), and V_E_ (ventilation, V_E_/VCO_2_ (equivalent for VCO_2_), and V_E_/VO_2_ (equivalent for VO_2_)).

The mean value of VO_2peak_ obtained during ITT was 3418.5 ± 585.1 mL·min^−1^ (50.2 ± 6.2 mL·kg^−1^·min^−1^), with men attaining 3732.1 ± 396.0 mL·min^−1^ (52.4 ± 5.2 mL·kg^−1^·min^−1^) and women 2728.6 ± 161.7 mL·min^−1^ (45.4 ± 6.0 mL·kg^−1^·min^−1^). The WLVO_2peak_ corresponded to 88.2 ± 13.7 N, 94.5 ± 11.2 N, and 74.3 ± 6.5 N for the group, for males, and for females, respectively. Figure 1 depicts the gas exchange response during the ITT and thresholds determination for a male swimmer. The criteria for maximal exertion during ITT were matched, since the respiratory exchange ratio (1.1 ± 0.1), HR (92.9 ± 4.2% HRmax), and blood lactate concentration (7.3 ± 1.4 mmol·L^−1^) all characterize a high-intensity aerobic exercise level. The profiles of VO_2_ and SR response during ITT followed a second-order polynomial pattern, as shown in Figure 2 (Panels A and B for a male swimmer, and Panels C and D for entire group responses). Among the swimmers, Fmean was 2.57 ± 0.58 N·kg^−1^ (2.73 ± 0.63 N·kg^−1^ for male and 2.20 ± 0.34 N·kg^−1^ for female swimmers).

The pulmonary gas exchange response during ITT is shown in Figure 2. The lower and upper limits for the isocapnic zone (GET and RCP) are clearly discernible from the responses of VE/VCO_2_ (Panel A), PetCO_2_ (Panel B), and VCO_2_ (Panel D), all in Figure 2. The GET attained 67.4 ± 7.3% of VO_2peak_ (males: 68.0 ± 8.0%; females: 66.0 ± 6.2%), and RCP was 87.4 ± 3.4% of VO_2peak_ (male: 87.5 ± 3.8%; female: 87.2 ± 2.9 %). The values of WLGET and WLRCP were 63.0 ± 3.7% and 85.2 ± 2.7% of WLVO_2peak_, respectively. For males, the values of WLGET and WLRCP reached 62.7 ± 4.3% and 85.2 ± 2.9% of WLVO_2peak_, and in females the WLGET and WLRCP were 63.6 ± 2.3% and 85.3 ± 2.7% of WLVO_2peak_, respectively.

The VO_2peak_ was attained during transition at WLVO_2peak_, as observed by the no significant difference to EEVO_2_ average values (*p* = 0.96) (Table 1). Therefore, swimmers attained VO_2peak_ during a constant-load test either directly from the response of the A_1′_ component or by the addition of the SC response. Just one female and three male swimmers showed no SC response, therefore reaching VO_2peak_ from the response of the A_1′_ component. The response of the A_1′_ component reached 91.6 ± 6.8% VO_2peak_ (males: 90.6 ± 7.7% VO_2peak_; females: 93.7 ± 4.0% VO_2peak_), with the remaining elevation of VO_2_ response until EEVO_2_ accounting for SC occurrence. The average tLim during the WLVO_2peak_ was 329.8 ± 63.6 s (male = 314.5 ± 66.8 s and female swimmers = 363.6 ± 44.0 s). In addition, the [La^−^] during the WLVO_2peak_ test reached average values of 7.4 ± 1.9 mmol·L^−1^ (males: 7.5 ± 1.8 mmol·L^−1^ and females: 7.4 ± 2.3 mmol·L^−1^), which did not differ from the [La^−^] value after ITT (*p* = 0.87); and the O_2_df average value was 1763.6 ± 714.1 mL·min^−1^ (males: 1987.1 ± 759.3 mL·min^−1^; females: 1270.2 ± 168.7 mL·min^−1^).

The EEVO_2_ showed positive correlations with VO_2peak_ and WLVO_2peak_ (r = 0.98 and 0.89; both at *p* < 0.01), as well as with O_2_df (r = −0.61; *p* = 0.01). Negative correlations were observed for t_Lim_ to VO_2peak_ (r = −0.55; *p* = 0.01), WLVO_2peak_ (r = −0.59; *p* < 0.01), GET (r = −0.58, *p* = 0.01), RCP (r = −0.53, *p* = 0.02), and EEVO_2_ (r = −0.50, *p* = 0.03). The level of correlations between t_Lim_ and the indexes of aerobic conditioning were associated with sample powers of 75, 82, 76, and 71%, respectively. Therefore, for the actual N = 16, there is a 25 and 18% chance of failing to detect an effect of VO_2peak_ and WLVO_2peak_ on t_Lim_. No other variable correlated to t_Lim_ at a significant level, despite SC and A_1′_ both showing a statistical tendency to correlate with t_Lim_ (r = −0.46 and 0.43, at *p* = 0.09 and 0.10, respectively).

**Figure 3:**
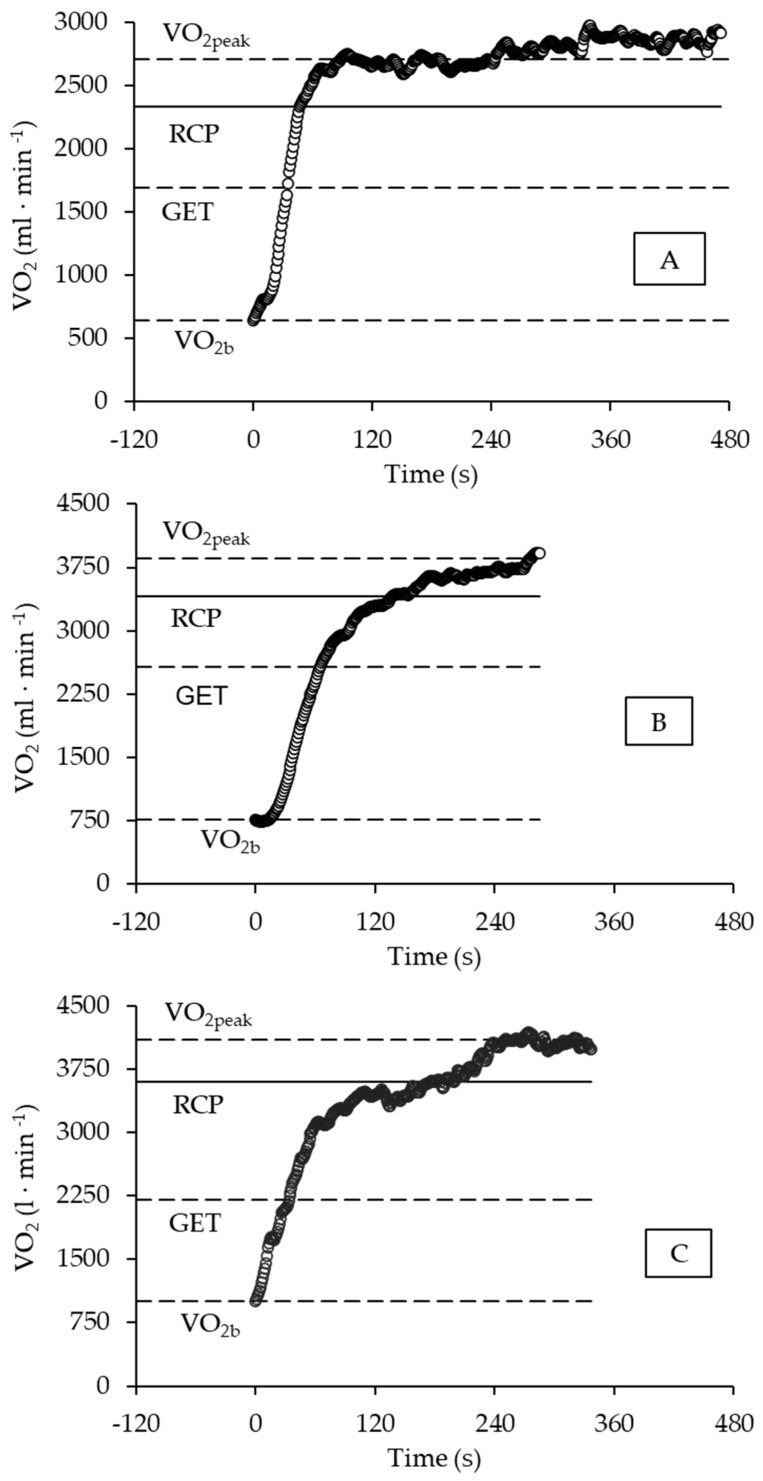
The profile of VO_2_ response during the WLVO_2max_ test. Panel (**A**) depicts a female swimmer, and Panels (**B**,**C**) show a male swimmer. See the detailed description in the text. Horizontal lines in each panel indicate (from the bottom to the top) the VO_2b_ (baseline VO_2_ response), GET (gas exchange threshold), RCP (respiratory compensation point), and VO_2peak_ (peak oxygen uptake).

The different profiles of VO_2_ and t_Lim_ responses during swimming performance at WLVO_2peak_ are depicted in Figure 3 (Panels A, B, and C). Panel A shows a female swimmer with long t_Lim_ (471 s), fast VO_2_ response (τ_1_ = 18.2 s), and reduced SC contribution (9.0%) to EEVO_2_. In Panel B is a male swimmer with short t_Lim_ (288 s), slow VO_2_ response (τ_1_ = 45.8 s), and average SC contribution (12.7%) to EEVO_2_; finally, in Panel C is a male swimmer with average t_L_im (337 s), slow VO_2_ response VO_2_ (τ_1_ = 30.6 s), and high SC contribution (18.8%) to EEVO_2_. For the swimmers in Panels A, B, and C, the [La^−^] was 6.7, 6.9, and 8.2 mmol·L^−1^, respectively.

## 4. Discussion

The findings corroborate that tethered swimming is suitable as an ergometer for the management of load intensity by means of the individual reference of maximal tethered force, from which a gradual metabolic demand was observed from submaximal to maximal rates with sufficient temporal resolution to identify GET, RCP, and VO_2peak_, as previously reported [9,13]. In addition, when performing at WLVO_2peak_, the VO_2_k response might be considered typical of a severe domain either in unimpeded front crawl swimming [14,15,18,20,21] or another exercise mode [30,35].

Furthermore, the t_Lim_ observed while performing at WLVO_2peak_ is in the range of the values reported for unimpeded front crawl swimming at maximal aerobic velocity (314 to 375 s) between swimmers with moderate VO_2peak_ [20,36]. However, even among elite swimmers with high VO_2peak_ (>70 mL·kg^−1^·min^−1^), the time limit values at maximal aerobic velocity presented a wide range (188 to 400 s) [21]. Moreover, evidence of the inverse association between time limit and maximal aerobic velocity, which was supported for cycling, running, swimming flume [37], and unimpeded front crawl swimming [25,37,38], with coefficients ranging from r = −0.47 to −0.72, was also found in the current study for tethered swimming. Additionally, the current study demonstrated an inverse association of t_Lim_ with other indexes of aerobic conditioning (such as VO_2peak_, GET, and RCP) and the VO_2_ elevation at the end of performance (such as EEVO_2_).

Notably, one of the physiological determinants of exercise tolerance in the severe domain is the aerobic conditioning level, which includes the central (i.e., rate of O_2_ availability) and peripherical (i.e., velocity of O_2_ phosphorylation) ability to control the adjustments of oxidative metabolism [23,39,40], and based on which higher and faster responses have been associated with shorter time limits in severe exercise during unimpeded front crawl swimming (r = −0.54 to −0.62) [21,37], cycling (r = −0.46) [23], and running (r = −0.75) [41]. Therefore, this assumption was also supported by the current findings, which contribute to reinforcing (from the negative association of t_Lim_ with VO_2peak_ and EEVO_2_) the need to consider other physiological aspects than the aerobic conditioning level to account for longer exercise tolerance in the severe domain.

In fact, exercising in the severe domain requires the gradual contribution of the finite anaerobic energy reserve in muscle fiber, probably due to the physiological constraints upon continuous increases in blood perfusion, gas diffusion, and mitochondrial function. This assumption associates exhaustion with metabolic acidosis and the depletion of intramuscular substrates [35,39,42,43], and therefore evidences the role of anaerobic capacity in time limit [23,39,40]. Particularly in swimming, another variable to consider is propelling efficiency, which can affect either the energy demand or the source of energy contribution [38,44].

Interestingly, there are still conflicting results on the role of propelling efficiency, as t_Lim_ has shown a wide range whatever the training level of swimmers [43,45], which was also evidenced in the current study with tethered swimming (197 to 496 s) when considering the t_Lim_ either between the sexes or for each sex as an independent group. In addition, higher boundaries for moderate and heavy domains (e.g., GET and RCP) showed to have a similar effect on time limit to the VO_2peak_ (i.e., shortening the time limit), of which comparable evidence was reported between the time limit and velocity at the anaerobic threshold (r = −0.54 to −0.62) for unimpeded front crawl swimming [21,37].

Thus, the most probable physiological scenario that might be associated with a longer time limit during severe exercise might be characterized by three main physiological responses assessed with the analysis of VO_2_k: (i) a fast time constant for primary amplitude (A_1′_) of the projecting VO_2_ close to the muscle demand, therefore avoiding a high O_2_ deficit at the beginning of exercise as well as stimulating anaerobic glycolysis early; (ii) enhanced control of the acid–base balance, preventing muscle and blood pH disturbance, as well as fast depletion of intra-muscular substrates; and (iii) ideally having a wide window for SC occurrence, allowing oxidative readjustments before being limited to the attainment of VO_2peak_, which is inevitable due to the progressive recruitment of fast glycolytic fibers [35,40,46]. The current study was pioneering in applying VO_2_k to the analysis of time limit during tethered swimming, from which three main responses were distinguished, as discussed below.

First, a longer time limit was observed for a female swimmer (Figure 3, Panel A), which exemplified the effect of the inverse relationship between time limit and WLVO_2peak_. Her values of WLVO_2peak_ (1.04 N·kg^−1^) and VO_2peak_ (36.4 mL·kg^−1^·min^−1^) were considered low when compared to the mean values for her sex-specific group. Despite being considered, therefore, a non-highly aerobic-conditioned female athlete, the high and fast VO_2_ primary response (i.e., amplitude, A_1′_; and time constant, t_1_) during the rest-to-exercise transition suggested no central or peripheral constrains to the oxidative raise until 99.7% of the predicted demand, hence avoiding earlier metabolic disturbance by reducing the O_2_ deficit, slow-component contribution, and blood lactate concentration. Thus, this physiological profile corroborated the assumptions that O_2_ diffusion, capillary perfusion, and mitochondrial function might be determinants of exercise tolerance among athletes with a moderate aerobic conditioning level when performing exercise in the severe domain [35,39,42].

In contrast, a second example observed was the short time limit for a male swimmer (Figure 3, Panel B), which might be an effect of the high values of WLVO_2peak_ (1.47 N·kg^−1^) and VO_2peak_ (58.8 mL·kg^−1^·min^−1^) when compared to the mean values of his sex-specific group. The negative effect on exercise tolerance might be accounted to the high target VO_2_ demand (high A_1′_) and the long time taken to be attained (i.e., slow time constant, τ_1_). Consequently, other physiological responses such as O_2_ deficit, slow-component contribution, and blood lactate concentration were prematurely enhanced. This situation is poorly tolerated by well-conditioned athletes due to the reduced anaerobic reserve [35,47], therefore corroborating the assumption that work muscle capacity is high among high-level athletes, in whom the ability to adjust the oxidative demand and delay the anaerobic activation to critical levels are limiting factors during high-intensity exercise [39,42].

Finally, the third profile of response observed for another male swimmer (Figure 3, Panel C) exemplifies the effect of superior anaerobic conditioning on time limit. Once again, a swimmer with no high aerobic conditioning level (WLVO_2peak_ = 1.03 N·kg^−1^, and VO_2peak_ = 47.6 mL·kg^−1^·min^−1^) showed reasonable tolerance (t_Lim_ > 300 s). The physiological profile accounting to this tolerance highlights the role of anaerobic capacity, as the slow-component contribution and blood lactate concentration should be high (i.e., ~19% and ~8 mmol·L^−1^, respectively), when the target primary VO_2_ demand attains a low rate (A_1′_~84% VO_2peak_) and its adjustment is similarly low (i.e., τ_1_~31 s) at the onset of exercise.

In fact, the relationship between the slow component and the cascade of physiological events leading to metabolic acidosis accounts for the activation of rapid glycolytic fibbers [35], which support the association between anaerobic capacity and longer time limit [23]. However, the present study observed no significant correlation between slow-component contribution with time limit or with blood lactate concentration, and therefore was closer aligned with studies showing the lack of correlation [20] than with studies reporting a positive correlation between the slow component and time limit [21,37]. Possibly, this physiological profile was a distinguishable response of the current sample of swimmers, but also indicates the particularity of the effect of the SC phenomena on time limit, which should further consider how each athlete adjusted and tolerated other physiological events taking place simultaneously [35,40].

However, the positive correlations between the slow component and the time limit has been evidenced for performance in maximal aerobic swimming velocity [21,37], suggesting that the larger the window for the SC manifestation, the greater the swimming tolerance should be at such swimming velocities, which is an assumption aligned to the aforementioned physiological profile of response at WLVO_2peak_. Although the SC is theoretically linked to the ability of fibers to further adjust to the VO_2_ demand, it is also a response linked with a concomitant increase in the reliance on anaerobic energy sources, which in turn can enhance energy cost and (probably) reduce tolerance among swimmers [20]. Therefore, most of the findings in the current study supported, or at least were aligned to, the metabolic profile of response reported for unimpeded swimming conditions.

However, the same swimmers were not evaluated in both swimming conditions. Thus, this is a limitation of the current study, and hence the direct comparison between both swimming conditions still remains to be analyzed in future studies, as well as whether the improvement of anaerobic conditioning by training with workloads corresponding to a severe domain has an effect on time-limited swimming performance. Moreover, we cannot attribute this physiological profile to a given particularity associated with sex and age group influence on performance ability during high-intensity swimming. For example, the (relative to body weight) energetic cost during short- and middle-distance swimming performance is not related to sex-specific differences in lean mass, nor does it have an influence on the slope (VO_2_ vs. velocity) of the incremental test in swimming [48]. Finally, there is evidence for the lack of influence of biological age on the association between short-distance swimming velocity and indexes of stroke mechanics and aerobic conditioning level [49], despite absolute (not relative) values of VO_2_ response showing the tendency to increase with biological age during a one-minute all-out bout of tethered swimming [50].

## 5. Conclusions

From the results of the incremental tethered test, the assessment of GET and the RCP by means of pulmonary gas exchange analysis was possible, and therefore it was possible to demarcate the domains for moderate, heavy, and severe exercise in tethered swimming conditions. Moreover, during the rest-to-exercise transition at a WL corresponding to VO_2peak_, it was possible to characterize three main profiles of metabolic processes underlying tolerance in a severe exercise domain by means of VO_2_ on-kinetics analysis. Indeed, the parameters of VO_2_k showed responses suggesting that tethered swimming might be reliable to simulate unimpeded front crawl physiological responses either at or around maximal aerobic velocity.

The findings also demonstrated that high tolerance was inversely related to the aerobic conditioning level observed for swimmers, independently of sex. In addition, the ability to adjust oxidative metabolism in order to match the target VO_2_ demand during exercise also reduced the time limit, which might contribute to increasing oxygen deficit. In turn, while the SC only tends to negatively affect the time limit, some individual responses suggested that this response might be dependent on the ability to tolerate high blood lactate accumulation. However, the magnitude and type of association between anaerobic capacity and time limit in the severe domain still remain to be addressed.

## Figures and Tables

**Table 1:** **Table 1**. The analysis of VO_2_k while performing tethered swimming at WLVO_2peak_.

	Group	Men	Women
VO_2b_ (ml·min^−1^)	665.8 ± 148.7	684.2 ± 146.5	625.2 ± 162.1
TD_1_ (s)	17.7 ± 5.1	17.9 ± 5.1	17.2 ± 5.5
τ_1_ (s)	24.4 ± 9.8	25.5 ± 11.7	22.2 ± 3.9
A_1′_ (ml·min^−1^)	3115.2 ± 497.4	3368.6 ± 360.7	2557.9 ± 193.6
R^2^	0.98 ± 0.02	0.97 ± 0.02	0.98 ± 0.00
TD_2_ (s)	149.3 ± 29.1	141.7 ± 26.5	166.0 ± 30.1
SC (ml·min^−1^)	333.6 ± 211.2	414.1 ± 215.6	172.6 ± 53.6
SC (%)	9.4 ± 5.1	11.0 ± 5.5	6.4 ± 2.4
EEVO_2_ (ml·min^−1^)	3427.6 ± 565.4	3744.5 ± 341.1	2730.5 ± 155.5
VO_2peak_ (%)	100.4 ± 3.8	100.6 ± 4.2	100.1 ± 3.1

VO_2Baseline,_ VO_2_ at baseline; TD_1_, time delay of the primary phase; τ_1_, time constant of the primary phase; A_1′_, amplitude of the primary phase; R^2^, R-squared; TD_2_, time delay of the slow component phase; SC, slow component; EEVO_2_, end-exercise oxygen uptake; VO_2peak_, peak oxygen uptake.

## Data Availability

The data that support the findings of this study are available from the last author (dalton.pessoa-filho@unesp.br), upon reasonable request.
